# Characterisation of the antiviral RNA interference response to Toscana virus in sand fly cells

**DOI:** 10.1371/journal.ppat.1011283

**Published:** 2023-03-30

**Authors:** Akira J. T. Alexander, Marco Salvemini, Vattipally B. Sreenu, Joseph Hughes, Erich L. Telleria, Maxime Ratinier, Frédérick Arnaud, Petr Volf, Benjamin Brennan, Margus Varjak, Alain Kohl

**Affiliations:** 1 MRC-University of Glasgow Centre for Virus Research, Glasgow, United Kingdom; 2 Department of Biology, University of Naples Federico II, Italy; 3 Department of Parasitology, Faculty of Science, Charles University, Prague, Czech Republic; 4 IVPC UMR754, INRAE, Univ Lyon, Université Claude Bernard Lyon1, EPHE, PSL Research University, Lyon, France; 5 Institute of Technology, University of Tartu, Tartu, Estonia; Division of Clinical Research, UNITED STATES

## Abstract

Toscana virus (TOSV) (*Bunyavirales*, *Phenuiviridae*, *Phlebovirus*, *Toscana phlebovirus*) and other related human pathogenic arboviruses are transmitted by phlebotomine sand flies. TOSV has been reported in nations bordering the Mediterranean Sea among other regions. Infection can result in febrile illness as well as meningitis and encephalitis. Understanding vector-arbovirus interactions is crucial to improving our knowledge of how arboviruses spread, and in this context, immune responses that control viral replication play a significant role. Extensive research has been conducted on mosquito vector immunity against arboviruses, with RNA interference (RNAi) and specifically the exogenous siRNA (exo-siRNA) pathway playing a critical role. However, the antiviral immunity of phlebotomine sand flies is less well understood. Here we were able to show that the exo-siRNA pathway is active in a *Phlebotomus papatasi*-derived cell line. Following TOSV infection, distinctive 21 nucleotide virus-derived small interfering RNAs (vsiRNAs) were detected. We also identified the exo-siRNA effector Ago2 in this cell line, and silencing its expression rendered the exo-siRNA pathway largely inactive. Thus, our data show that this pathway is active as an antiviral response against a sand fly transmitted bunyavirus, TOSV.

## Author summary

Toscana virus (TOSV) is a bunyavirus that is transmitted to humans through the bite of phlebotomine sand flies. Examining the immune responses that control arboviruses in their vectors is critical for understanding how these organisms control infection, as this may affect transmission. The exogenous siRNA (exo-siRNA) pathway is a critical antiviral immune response in mosquitoes, but much less is known about the pathway in sand flies. Here we show that a functional exo-siRNA pathway exists in a sand fly-derived cell line and that it is active as a response to TOSV infection. These findings improve our understanding of antiviral immunity in vectors in general, and phlebotomine sand flies in particular.

## Introduction

Toscana virus (TOSV) (*Bunyavirales*, *Phenuiviridae*, *Phlebovirus*, *Toscana phlebovirus*) is transmitted by dipteran, blood feeding phlebotomine sand flies (*Psychodidae*) in countries around the Mediterranean Sea, and the virus is increasingly also detected beyond. TOSV-induced disease often manifests as mild febrile illness in humans, but cases of meningitis and encephalitis have been described which sets it apart from other sand fly transmitted phleboviruses. Several sand fly species have been implicated, or suggested as vectors of TOSV, with *P*. *perniciosus* and *P*. *perfiliewi* relevant for transmission to humans. Three lineages of TOSV (A, B, C) have been described although C has not yet been isolated as infectious virus [1,2].

Like that of other phleboviruses [3], the genome of TOSV consists of negative sense (L, M segments) or ambisense (S segment) RNA strands. The L (large) segment encodes the RNA-dependent RNA polymerase, the L protein; the M (medium) segment encodes the structural glycoproteins Gn/Gc and the non-structural NSm protein. The ambisense S (small) segment encodes the nucleocapsid (N) and NSs proteins. Replicative functions are carried out by the L and N proteins. The NSs proteins of phleboviruses carry out critical functions as type I interferon response antagonists in vertebrate cells [4]. Recently, reverse genetics systems for TOSV A and B have been described [5,6], which opens new possibilities for studies of these pathogens.

Relatively little is known about the immune response induced by arboviruses in sand flies. In mosquitoes, a major group of insect vectors of arboviruses, RNA interference (RNAi) pathways are critical for controlling virus replication. Of which, the exogenous siRNA (exo-siRNA) pathway plays a key role in antiviral responses. While some steps in these processes are not yet clear, the strong levels of homology with this pathway in the model organism *Drosophila melanogaster* suggests that critical mechanisms are present across insects, demonstrated by a high degree of conservation amongst RNAi effectors, including Dicer and Argonaute proteins. Virus replication induces dsRNA, which is sensed by the nuclease Dcr2 and cleaved into predominantly 21 nucleotide (nt) virus-derived small interfering RNAs (vsiRNAs). These vsiRNAs are loaded into Ago2, which is part of the RNA induced silencing complex (RISC). One strand of the vsiRNA is retained and used to target and degrade complementary sense viral RNA. This process is generally well conserved across arthropods and is required to control arbovirus replication in mosquito cells [7–10].

The 21 nt vsiRNAs produced in response to arboviral infection usually map over the entire length of the viral genome and antigenome, and production occurs in a processive manner by Dcr2 at least during Semliki Forest virus (SFV, *Togaviridae*) infection of an *Ae*. *aegypti* derived cell line [11]. A further group of small RNAs induced by arbovirus infection called virus derived PIWI interacting RNAs (vpiRNAs) have a size distribution of 24–30 nt and a signature motif (U at position 1, and A at position 10) [12]. For a phlebovirus related to TOSV, mosquito-borne Rift Valley fever virus (RVFV), the induction of 21 nt vsiRNA as well as vpiRNAs has been shown during RVFV infection of mosquito cell cultures, with antiviral roles demonstrated for the exo-siRNA pathway as well as the Piwi4 protein [13,14]. In comparison to mosquitoes much less has been reported regarding the RNAi responses of sand flies to viral infection. The existence of nucleic acid induced, non-specific antiviral responses in cells of the *Leishmania* vector *Lutzomyia longipalpis* has been demonstrated previously [15,16]. A recent study investigating *in vivo* and *in vitro* infections of *L*. *longipalpis* with vesicular stomatitis virus (VSV) showed the production of 21 nt vsiRNAs, but intriguingly not vpiRNAs, suggesting differences in pathway induction to mosquitoes. Nonetheless, silencing Ago2 expression in this system led to an increase in viral RNA, thus demonstrating the relevance of the exo-siRNA pathway in controlling viral infections [17]. VSV is however not usually transmitted by sandflies [18] and perhaps virus-dependent mechanisms also influence antiviral RNAi responses.

Here we set out to assess the presence of an antiviral RNAi response in a *P*. *papatasi*-derived cell line (PP9) and its effect on bunyavirus replication. We adapted this cell line -PP9ad- for improved transfection, and using reporter assays, confirmed the presence of an exo-siRNA pathway activity in these cells. Infection with TOSV resulted in the production of 21 nt vsiRNA that clustered along the viral genome and antigenome. We also noted that no vpiRNAs were detected in response to TOSV infection. In addition, we identified the Ago2 coding sequence of these cells, and showed that it is required for exo-siRNA pathway activity. The findings here show new possibilities for the study of RNAi in sand flies and derived cell lines and allows comparisons of RNAi responses to other arthropod vectors of arboviruses.

## Materials and methods

### Cell culture and viruses

Cells of vertebrate origin used in this study were human A549 NPro (originally called A549/BVDV NPro expressing bovine viral diarrhoea virus NPro protein; a kind gift of R. Randall, University of St. Andrews, UK) [19] and hamster BSR-T7/5 CL21 [20]. A549 NPro cells were grown in DMEM (Gibco, Waltham, MA, USA) supplemented with 10% fetal bovine serum (FBS; Gibco, Waltham, MA, USA) and 1000 units/ml penicillin and 1 mg/ml streptomycin (P/S; Gibco, Waltham, MA, USA) supplemented with blasticidin (InvivoGen, San Diego, CA, USA) at 10 μg/ml. BSR-T7/5 CL21 cells were grown in GMEM (Gibco, Waltham, MA, USA) supplemented with 10% FBS,10% tryptose phosphate broth (TPB; Gibco, Waltham, MA, USA), P/S and additionally supplemented with 0.25 mg/ml G418 (Sigma-Aldrich, St. Louis, MO, USA). All vertebrate cell cultures were maintained at 37°C and 5% CO_2_. *P*. *papatasi*-derived PP9 cells were obtained from the World Reference Center for Emerging Viruses and Arboviruses (University of Texas Medical Branch, Galveston, Texas, USA). These cells were derived from embryonated eggs, using methods previously described [21,22]. PP9 cells were grown in Mitsuhashi & Maramorosch (M & M) medium (CaCl_2_ · 2H_2_O 250 mg/l, MgCl_2_ · 6H_2_O 125 mg/l, KCl 250 mg/l, NaHCO_3_ 150 mg/l, NaCl 8750 mg/l, H_2_NaO_4_P 250 mg/l, d(+) C_6_H_12_O_6_ 5000 mg/l, lactalbumin hydrolysate 8125 mg/l, yeastolate 6250 mg/l) supplemented with 20% FBS and P/S. PP9ad cells (adherent cells developed from PP9 cells, developed in this study as described in S1 Method) were grown in Leibovitz L-15 medium (Gibco, Waltham, MA, USA) supplemented with 10% FBS, 5% TPB, 1 X L-Glutamine (Gibco, Waltham, MA, USA) and P/S. All insect cell cultures were maintained at 28°C without supplemental CO_2_.

TOSV lineage A virus (rTOSV) produced by reverse genetics using BSR-T7/5 CL21 cells (previously described in [6]) was used for infection studies. To create stocks of rTOSV, the medium from 6-day old transfected BSR-T7/5 CL21 cell lines was clarified by centrifugation before 100 ul was added to a 150 cm^2^ flask of A549 NPro cells in DMEM containing 2% FCS. Virus stocks were incubated at 33°C, 5% CO_2_ for 7 days, before media were collected, clarified and viral particles concentrated in a 100,000 kDa protein concentration spin column. The resulting concentrate was resuspended in fresh medium to a final volume of 20 ml, the pH adjusted using Sodium Bicarbonate and stored at—80°C.

rTOSV was titrated by plaque assay on A549 NPro cells under an overlay of 1X MEM, 2% newborn calf serum (NBCS; Gibco, Waltham, MA, USA) and 0.6% Avicel (FMC Biopolymer, Philadelphia, PA, USA). These were incubated at 37°C and 5% CO_2_ for 5 days. Infected cell monolayers were then fixed in 4% formaldehyde and stained with trypan blue for visualisation. All viral titres referred to are given as plaque forming units/ml (PFU/ml).

### Western blotting

Cells were lysed in passive lysis buffer (Promega, Madison, WI, USA) and samples reduced before being separated on a 10% Bis-Tris polyacrylamide gels (Invitrogen, Waltham, MA, USA). Anti TOSV-N-MA29010 [6] was used at a final dilution of 1:1000 and incubated overnight at 4°C. A loading control of anti α-tubulin (#T5168, Sigma-Aldrich, St. Louis, MO, USA) was used at a final dilution of 1:5000. Images were obtained on a LI-COR Odyssey CLX far red imaging system and analysed using LI-COR Image Studio LI-COR Biosciences (Lincoln, NE, USA).

### Reference transcripts

A *de novo* transcriptome assembly was produced using 49 available *P*. *papatasi* paired-end RNA-seq data sets (Accession Numbers: ERR3714215 to ERR3714262, ERR3714265 and ERR3714266) available at the Sequence Read Archive and European Nucleotide Archive. All data sets were concatenated into two paired FASTQ files, and *de novo* assembled using the Trinity software (release v2.5.1) [23,24] with default parameters on the ADA Server at the Department of Biology, University of Naples Federico II (24 cores, 256 GB of memory). The resulting transcriptome consists of 252,949 assembled transcripts with a N50 value of 794. The assembled *P*. *papatasi* transcriptome was searched with the TBLASTN algorithm using *in silico* probes of the putative Ago2 protein sequences of *P*. *perniciosus* [25]. *P*. *perniciosus* and *P*. *papatasi* Ago2 alignments were produced using Clustal Omega online tool (https://www.ebi.ac.uk/Tools/msa/clustalo/).

### 5’ RACE

5’ RACE was carried out using the NEB Template Switching RT (New England Biolabs, Ipswitch, MA, USA) kit according to the manufacturers protocol. cDNA was generated using oligo dT or the gene specific primer AGO2_5’RACE_Rv with the DNA/RNA hybrid template switching oligo (TSO), [GCTAATCATTGCAAGCAGTGGTATCAACGCAGAGTACAT]rGrGrG. The 5’ end of the transcript was amplified using a primer underlined in the TSO above and an Ago2 specific reverse primer. The PCR products were sequenced by Sanger sequencing as before at 100 bp resolution. Primers are listed in S1 Table.

### Ago2 sequencing

RNA was extracted from PP9ad cells using Trizol (Invitrogen, Waltham, MA, USA) and used to generate cDNA with both gene specific primers based on transcriptomic data and oligo(dT) using Superscript II RT polymerase (Invitrogen, Waltham, MA, USA). Sequences encoding the putative Ago2 protein were amplified from cellular cDNA using a series of primers spanning overlapping 1kb sections. Simultaneous reactions also amplified the full-length transcript. All products were then sequenced using Sanger sequencing (Eurofins Genomics, Wolverhampton, England, UK).

### Sequence alignments

Verified arthropod Ago2 sequences were downloaded from NCBI and Vectorbase. These were then aligned using CLC Genomics Workbench 7 (Qiagen, Hilden, Germany) with the sequence generated from PP9ad cells. The alignment was used to generate a neighbour joining tree using the Jukes-Cantor substitution model. This was bootstrapped with 500 replicates; the bootstrap values are shown as percentages.

### Small RNA sequencing

For each condition -mock vs infected- three wells of 5 x 10^6^ PP9ad cells were infected with rTOSV at an MOI of 10 or treated with plain medium for the control and incubated for 3 days at 28°C. At the given timepoint, RNA was then extracted from mock- or TOSV-infected wells using Trizol following the manufacturers protocol and each corresponding triplicate sample was pooled. This was then repeated a further two times to generate three independent replicates. Small RNA sequencing was carried out by the Beijing Genomics Institute (BGI Group, Shenzhen, China). Extracted RNA was size separated using polyacrylamide gel electrophoresis (PAGE) and bands in the region of 18–35 nt extracted. These had a 3’ and 5’ adaptor added before amplification by RT-PCR. After purification of the PCR products, they were heat denatured and circularised using a splint oligo. The single stranded circular DNA became the library. Library QC was carried out using an Agilent Technologies 2100 bioanalyser (Agilent Technologies, Santa Clara, CA, USA). The circular library was PCR amplified using phi29 polymerase to form DNA nanoballs (DNB). The DNBs were loaded onto a patterned nanoarray, and single end 50 base reads generated by combinatorial probe anchor synthesis using a DNBSeq-G400 sequencer (MGI Tech. Co., Shenzhen, China). This generated 29,726,412 reads for MOCK_1, 29,422,851 reads for MOCK_2, 29,557,706 reads for MOCK_3, 29,357,786 reads for rTOSV_1, 29,459,534 reads for rTOSV_2 and 29,316,585 reads for rTSOV_3. Data were deposited in the NCBI SRA (PRJNA879406).

### RNA sequencing analysis

The high throughput RNA sequencing data was analysed as reported earlier [26,27]. The short read quality control program Trim galore (https://www.bioinformatics.babraham.ac.uk/projects/trim_galore/) was used to remove low quality (Phred score 30) and short (<18 nt) reads from sequencing data. Following that, reads were mapped to the reference genome sequence (GenBank MT032308, MT032307, MT032306 for L, M, and S segments, respectively), with a maximum of one mismatch or indel allowed. Subsequently, reads with alignment lengths ranging from 18 to 35 nt were divided into two groups based on their mapping orientation, which was either positive (antigenomic) or negative (genomic) sense.

Further analysis was carried out using CLC Genomics, RStudio (RStudio, Boston, MA, USA) or Excel in the Office 365 package (Microsoft, Redmond, WA, USA). All data sets were scaled as a proportion of whole to remove differences in overall read quantities before analysis. All data is displayed as such in the figures. To calculate the coverage, each individual 21 nt vsiRNA was turned into an array of 21 numbers with the first number in the array being the start position. The coverage was then calculated by quantifying the number of times a genome nucleotide position was included in a 21 nt vsiRNA array.

### dsRNA production

dsRNA was created using PCR derived templates with a 5’ and 3’ T7 RNA polymerase promoter sequence using the MegaScript RNAi kit (Invitrogen, Waltham, MA, USA). The dseGFP template was amplified from phPGK-eGFP and the dsFFLuc template was amplified from pGL3-PUb [28]. dsRNA targeting Ago2 was generated from cDNA in a similar manner.

### Transfection, dsRNA transfection, luciferase assays and rTOSV infection

For DNA transfection, 5 X 10^5^ cells/well of the PP9 or PP9ad cell line were transfected with 500 ng plasmid using transIT-LT1 at 3 ul reagent per 1 ug of DNA. For dsRNA containing transfections, 5 X 10^5^ cells/well of the PP9ad cell line were transfected with 100 ng pGL3-PUb (expressing firefly luciferase, FFLuc, under control of the *Ae*. *aegypti* polyubiquitin promoter PUb) [28], 7 ng pPUb-RLuc (produced in this study, expressing *Renilla* luciferase [RLuc]) and 20–100 ng of dsRNA using Dharmafect 2 (Horizon Discovery Ltd, Waterbeach, England, UK) according to the manufacturer’s protocol. Transfected cell cultures were lysed 48 h post transfection (h.p.t.) in passive lysis buffer and luciferase measured using the Dual Luciferase Assay System (Promega, Madison, WI, USA). For dsAgo2 assays, transfection of the first set of dsRNAs occurred 24 h before the cells were treated as above. For the rTOSV infection assays, dsRNA was transfected as before, 24 h prior to infection with rTOSV.

### Generation of pPUb-RLuc

For the production of pPUb-RLuc, *Renilla* luciferase was amplified using PCR from pRL-TK (Promega, Madison, WI, USA) and inserted into pPUb [29] using InFusion assembly (TaKaRa Bio, USA) to create the reporter plasmid. The PUb (polyubiquitin) promoter from *Ae*. *aegypti* was described previously [28].

### RT-qPCR analysis

RNA was extracted from PP9ad cells using Trizol 48 h.p.t. of dsRNA. cDNA was generated from the mRNA using oligo d(T)_15_ (New England Biolabs, Ipswitch, MA, USA) and SuperScript III RT polymerase (Invitrogen, Waltham, MA, USA) according to the manufacturer’s protocols. qPCR was performed using Fast SYBR Green (Applied Biosystems, Waltham, MA, USA) and the Quantstudio 3 RT-PCR system (Thermo Fisher, Waltham, MA, USA). The superoxide dismutase (*sod*) gene was used for normalisation of results [25]. Relative gene expression was calculated as described [30].

### Statistical analysis

Statistical analysis was performed using Minitab v19 (Minitab LLC, State College, PA, USA). Data underlying figures are available under http://dx.doi.org/10.5525/gla.researchdata.1373.

## Results

### Adaptation of the PP9 cell line for experimental use

**Fig 1:**
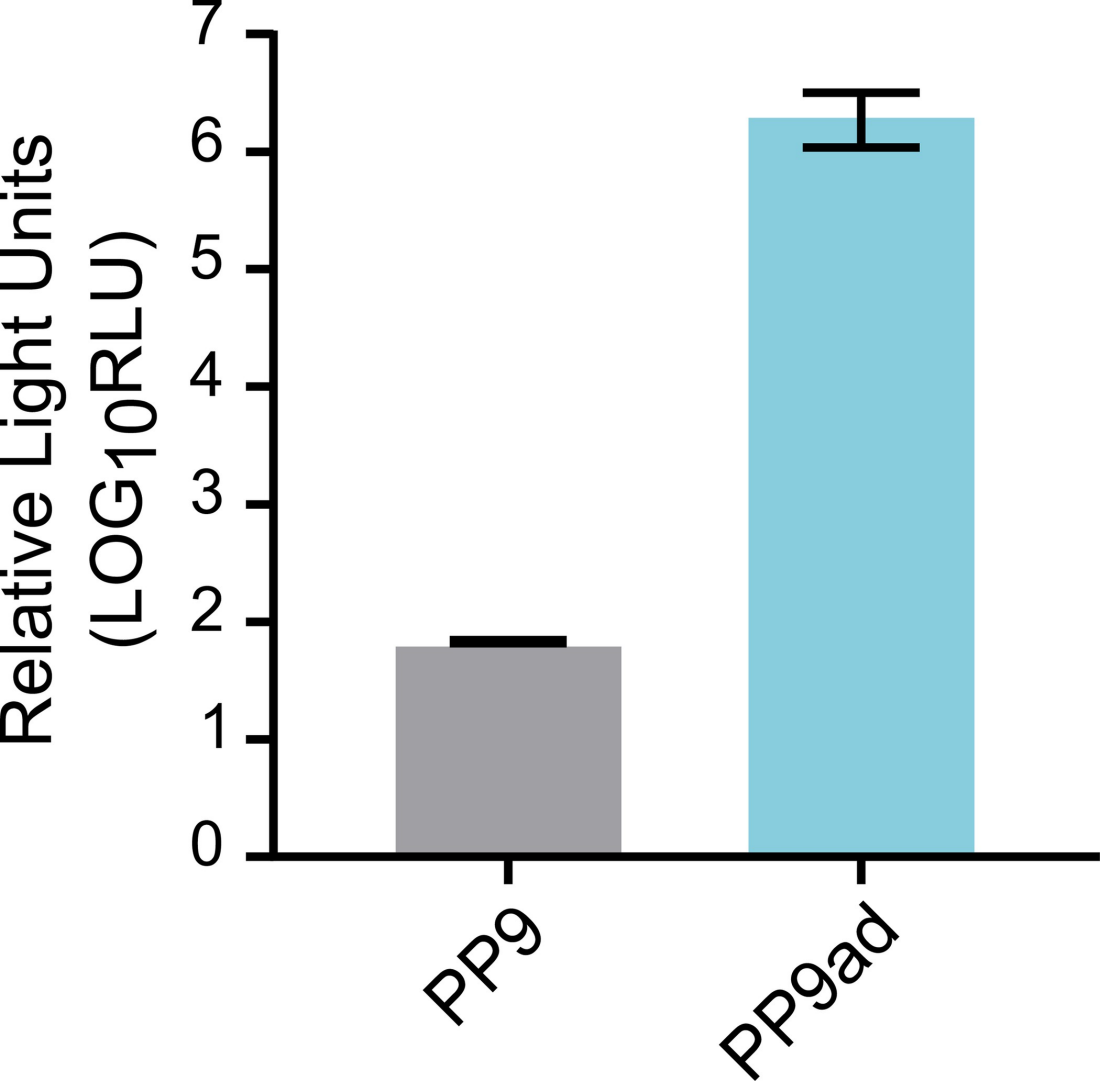
FFLuc activity in transfected PP9 versus PP9ad cell lines. PP9 and PP9ad cells were transfected with pGL3-Pub. At 72 h.p.t., cells were harvested and FFLuc activity was measured and normalized to untransfected. Shown is the relative light output from pGL3-PUb transfected cells, using TransIT-LT1. The figure shows data from three independent experiments, each conducted with three technical replicates. Data presented reflect mean values and error bars depict standard error of the mean (SEM).

The original PP9 cell line as received, was found to be unamenable to transfection. This is believed to be partly due to issues of culture and poor adherence to cell culture plasticware. Adherent cells were selected by repeated serial passage with non-adherent cells removed through medium change. The optimum media conditions were improved through omission/addition assays, eventually leading to the use of L-15 medium with supplements, as described in Materials and Methods. The resulting adapted cell line was termed ‘PP9ad’. These cells were amenable to transfection using Mirus TransIT-LT1 to deliver a construct containing FFLuc under the control of the *Ae*. *aegypti* PUb promoter (pGL3-PUb). Transfection of pGL3-PUb into PP9ad resulted in a strong FFLuc luciferase signal, compared to that detected in the original PP9 cell line (Fig 1).

**Fig 2:**
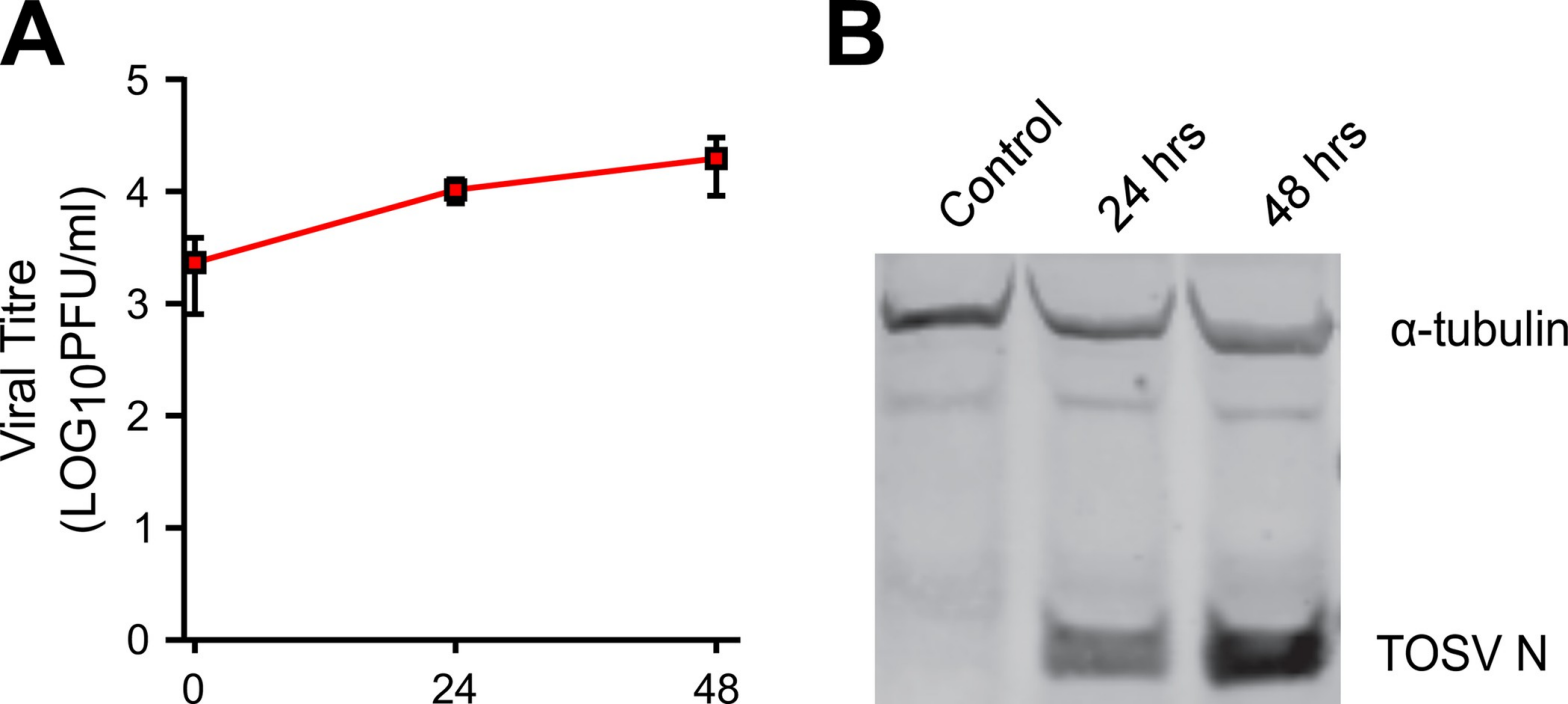
The PP9ad cell line is susceptible to rTOSV infection. **(A)** Titration of rTOSV following PP9ad infection with rTOSV (MOI 5 PFU/cell) up to 48 h post infection (h.p.i.). Mean titre with standard deviation (SD) of three samples are presented. **(B)** Protein lysates from rTOSV-infected PP9ad cells were assessed for the presence of N protein (TOSV N) by western botting at 24 and 48 h.p.i. ⍺-Tubulin was used as a loading control. Figures are representative of two independent experiments; lysate from uninfected cells was used as negative control.

PP9ad cells were infected with rTOSV at a multiplicity of infection (MOI) of 5 PFU/cell. Progression of infection was monitored by plaque assay titration of virus released into the cell culture supernatant and western blot detection of N protein accumulation in infected cells (Fig 2). A modest increase in viral titre as well as increase in N protein levels were confirmed. Thus, the PP9ad cell line supports rTOSV replication.

### Identification of Ago2 in PP9ad cells

**Fig 3:**
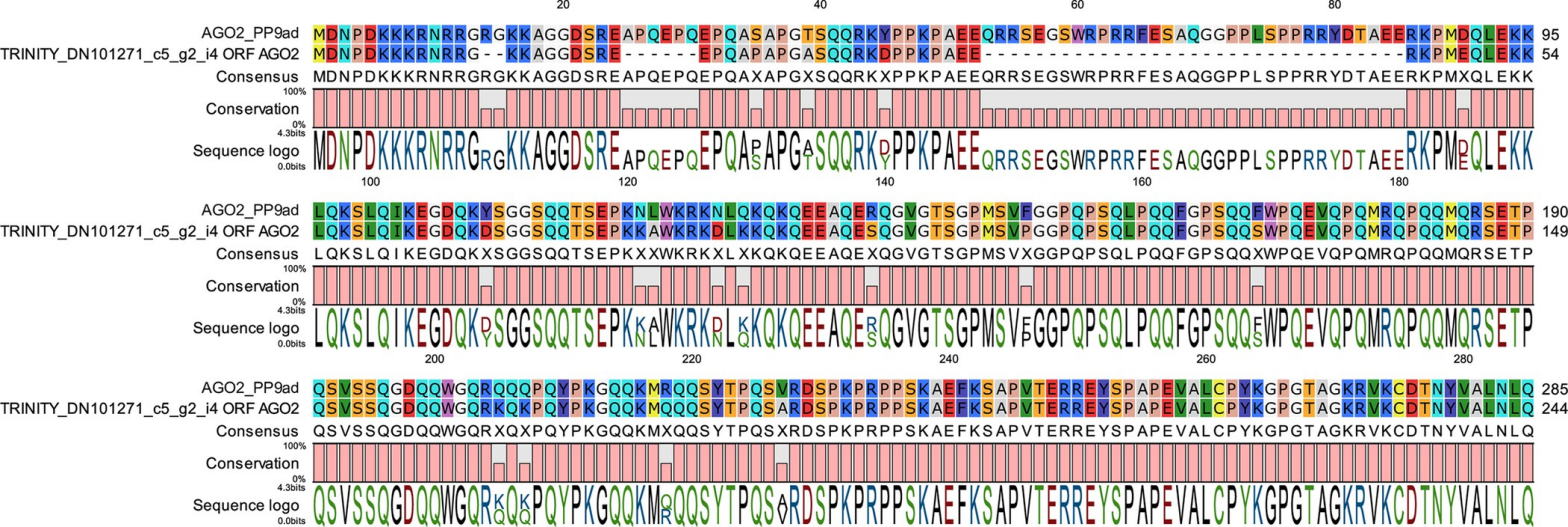
Consensus between translated *P*. *papatasi* transcriptomics derived and PP9ad cell Ago2 sequences. The Ago2 amino acid sequences differed in the N terminal region mainly, as shown in partial sequence indicated; the PP9ad cDNA encoding a longer protein.

A putative sequence for Ago2 was identified in the TRINITY_DN101271_c5_g2_i4 transcript from transcriptomics analysis of adult *P*. *papatasi* flies. Primers to amplify the putative Ago2 sequence were generated and following reverse transcription, the resultant cDNA product from PP9ad cells was sequenced. The 5’ region of the cDNA sequence did not map to the reference however, so further primers were used to generate cDNA from this region. cDNA synthesis, sequencing, and alignment were repeated until a final consensus was created. A small region from 157–255 nt could not be resolved, with a longer sequence appearing in the PP9ad cells. To resolve the 5’ region, 5’ RACE was carried out and the 5’ region amplified by PCR and sequenced. This confirmed the presence of 99 nt of sequence in PP9ad cells that was not present in the transcript described above (Fig 3). The final sequence was deposited with GenBank under accession number OP744464.

**Fig 4:**
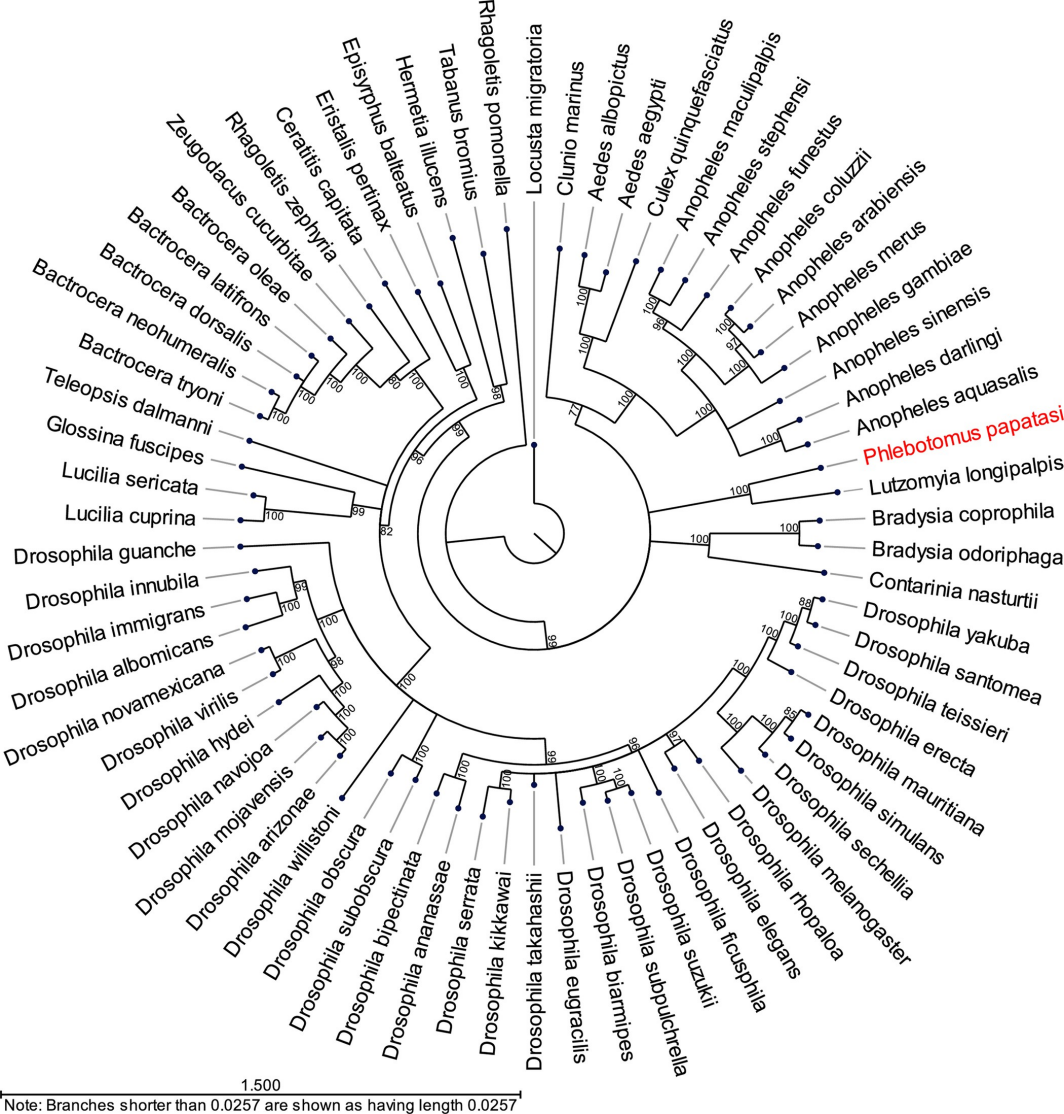
Neighbour-joining tree of dipteran Ago2 protein sequences. Full length Ago2 sequences were obtained for the Order *Diptera* from NCBI (see S2 Table for accession numbers) and aligned with the PP9ad-derived Ago2 consensus sequence. The phylogeny was reconstructed using the neighbour-joining method and Jukes-Cantor substitution model and rooted on *Locusta migratoria*. The phylogram is the result of 500 bootstrap replications. The branch lengths are drawn to scale and the scale bar in the bottom left represents 1.5 substitutions per site.

The protein sequence of Ago2 identified from the PP9ad cell line was compared to full-length dipteran Ago2 sequences available at NCBI, and a neighbour-joining phylogram constructed (Fig 4). The *P*. *papatasi* Ago2 sequence identified from PP9ad cells was found to be most similar to an Ago2 sequence derived from the *L*. *longipalpis* sand fly, followed by other Diptera *spp*. like mosquito, midge and gnat derived Ago2 sequences. Accession numbers used in this figure are listed in S2 Table.

### PP9ad cell lines contain a functional exo-siRNA pathway which is dependent on Ago2 activity

**Fig 5:**
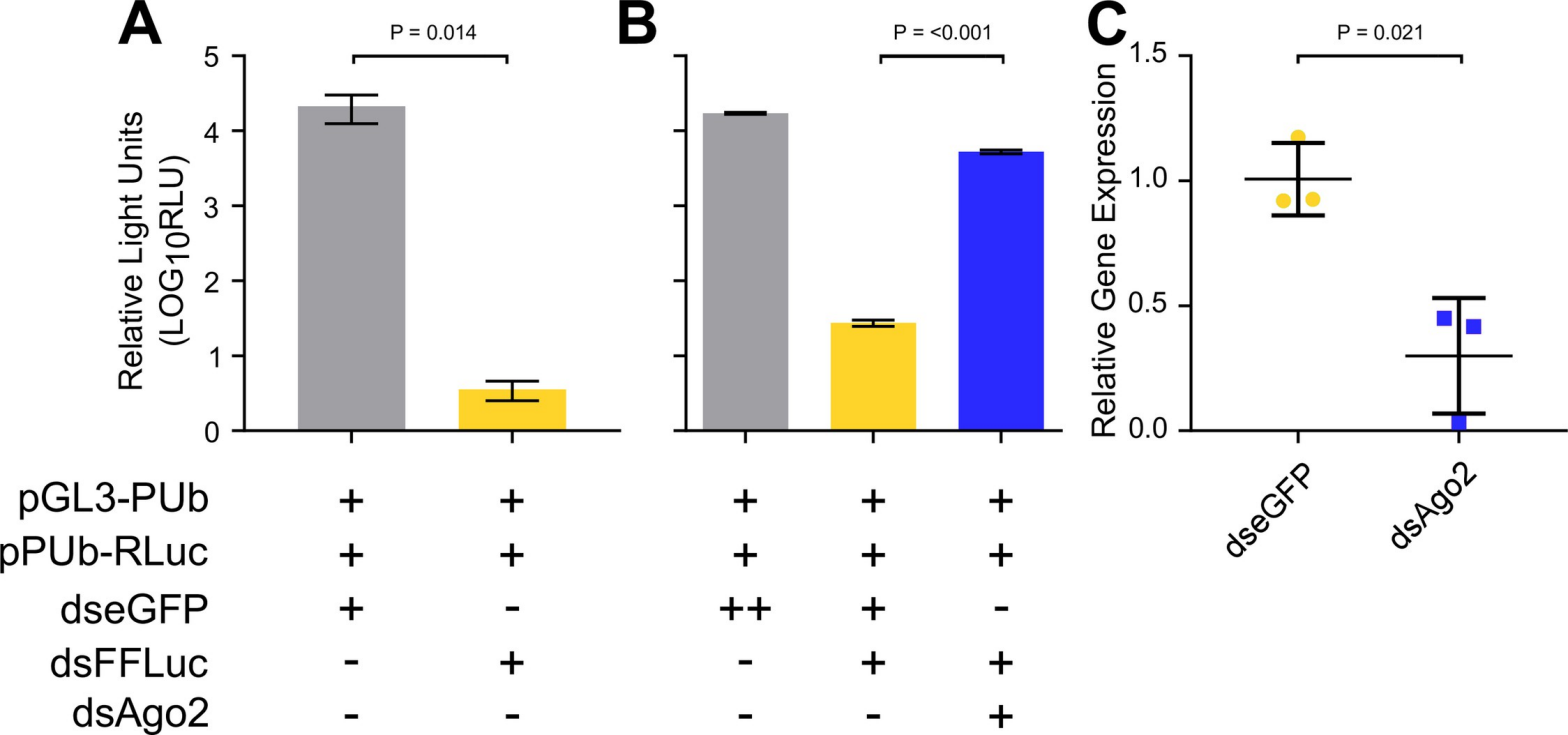
The PP9ad cell line contains an exo-siRNA pathway. PP9ad cells were transfected with a combination of reporter plasmids and dsRNAs, and harvested for reporter expression by luciferase assay, or to determine gene expression by quantitative PCR. **(A)** Effects on normalised FFLuc expression in PP9ad cells when transfections of reporter plasmids were supplemented with 100 ng dsRNA targeting eGFP (dseGFP) or FFluc (dsFFLuc). FFLuc expression was normalized to a RLuc internal control (unpaired t test, T = 4.21, P = 0.014). **(B)** PP9ad cells were transfected with either 100 ng dsRNA targeting eGFP (dseGFP) or Ago2 (dsAgo2) 24 h prior to transfection with pGL3-PUb and pPUb-RLuc expression plasmids and 20 ng dseGFP or dsFFLuc as indicated (ANOVA, F = 1869.21, P = <0.001). FFLuc expression was normalized to a RLuc internal control. **(C)** The relative abundance of Ago2 present in PP9ad cells transfected with either 100 ng dseGFP or dsAgo2 was determined. Cells were harvested and relative abundance of Ago2 determined by RT-qPCR at 48 h.p.t. (unpaired t test, T = 4.48, P = 0.021). Data are representative of three independent replicates and presented as the mean value ± SD.

Following the successful identification of the PP9ad Ago2 sequence and the optimization of transfection conditions, the presence of an exo-siRNA response in PP9ad cell lines was tested. Initially PP9ad cells were transfected with a combination of pGL3-PUb, pPUb-RLuc and either dsRNA against eGFP or FFLuc. Following transfection of dsRNA targeting FFLuc (dsFFLuc), we observed a reduction in FFLuc signal, compared to control conditions where a dsRNA to eGFP was added. This indicated a robust and specific silencing of targeted gene expression in PP9ad cells in response to addition of the corresponding dsRNA (Fig 5A).

Next, we wanted to ascertain if the silencing reported in Fig 5A was mediated by Ago2. A dsRNA for silencing of PP9ad-derived Ago2 was designed to target a region between nt position 273 and 872 at the 5’ end of the Ago2 transcript. Transfection of 20 ng of dsFFLuc was sufficient to achieve abolishment of the FFluc signal. However, when dsFFLuc was transfected with 100 ng of dsAgo2, FFLuc measurements increased compared to the dsFFluc-/dseGFP-transfected control and to similar levels recorded in the dseGFP-/dseGFP- transfected negative control (Fig 5B). These data show that the exo-siRNA pathway could be triggered by dsRNA in PP9ad cells and that its function was dependent on the activity of Ago2. Next, we quantified the relative levels of Ago2 mRNA after silencing using qRT-PCR and noted a significant reduction in the amount of Ago2 mRNA present in the PP9ad cells following transfection with dsAgo2 RNA (Fig 5C), confirming that knock down was effective.

**Fig 6:**
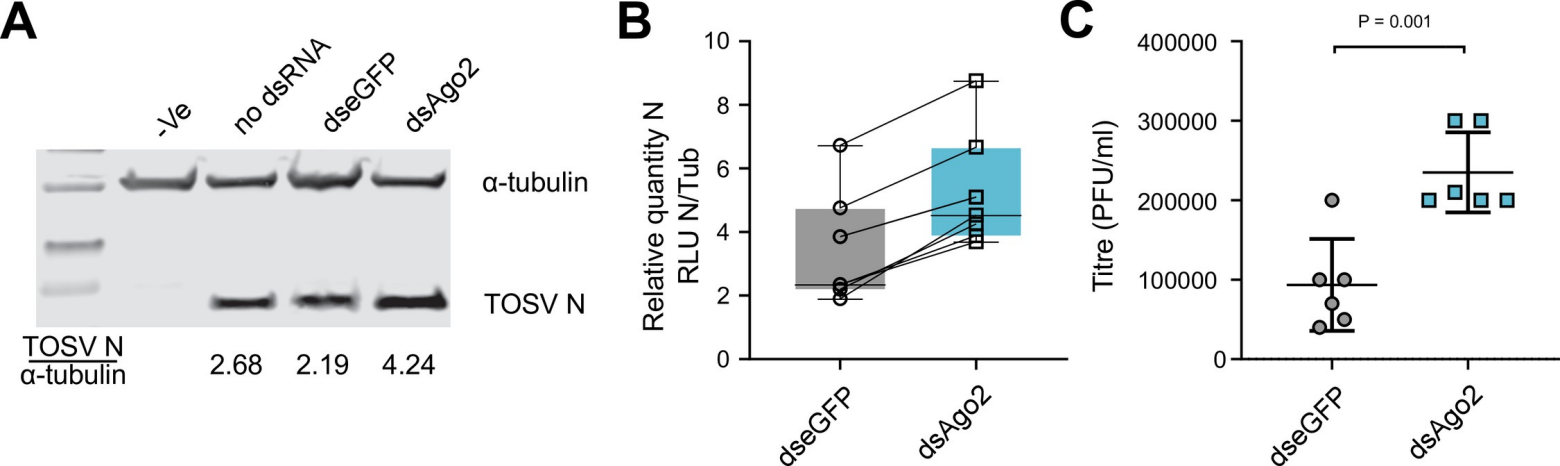
Depletion of Ago2 in rTOSV-infected PP9ad cell cultures leads to an increase in virus replication. PP9ad cells were either mock transfected or transfected with 100 ng of dseGFP or dsAgo2 for 24 h before being infected with rTOSV at a MOI of 10 PFU/cell. At 48 h.p.i., cells were harvested. **(A-B)** Cell lysates were probed with anti-TOSV N and anti-⍺-tubulin antibodies, the ratio of TOSV N RLU:⍺-tubulin RLU was calculated and presented for the dseGFP and dsAgo2 samples. **(C)** Viral titres in cell culture supernatants were titrated by plaque assay. Data analysed by unpaired t-test, P = 0.001 (C). Figure shows data from seven independent blots (B) and six independent infections for titrations (C), data are presented as the mean value ± SD.

To determine if Ago2 has an antiviral effect on the replication of rTOSV in the PP9ad cells, we infected cells after transfection with dsRNA targeting Ago2. dsAgo2 transfected PP9ad showed on average twice the level of detectable rTOSV N protein at 48 h.p.i. compared to control infections (Fig 6A and 6B) and we observed a small but significant increase in viral titre (Fig 6C).

### The PP9ad cell line produces 21 nt vsiRNAs in response to rTOSV infection

**Fig 7:**
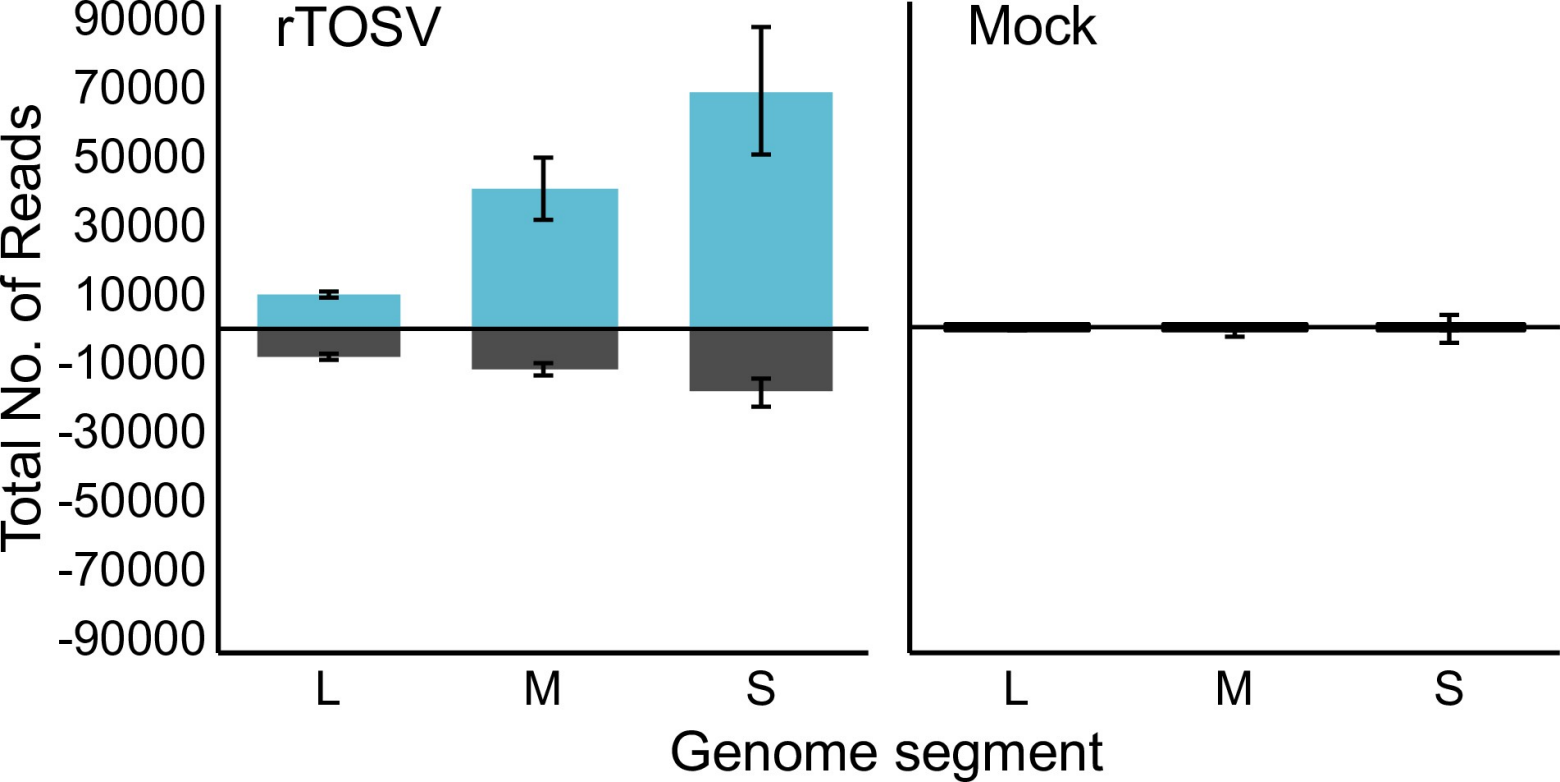
Overall coverage of small RNAs mapping to rTOSV segments. The PP9ad cell line was infected with rTOSV at a MOI of 10 PFU/cell or mock infected. At 3 d.p.i., cells were harvested and small RNAs were isolated and the overall distribution of small RNA sequences mapping to the genomic (black, negative reads) or antigenomic (blue, positive reads) viral S, M and L RNA segments. Data presented as mean ± SD from three independent experimental repeats.

To determine the ability of the PP9ad cell line to mount an RNAi response to virus infection, the presence of virus derived small RNAs was assessed 3 days post infection (d.p.i.) with rTOSV at a MOI of 10 PFU/cell. Infected PP9ad and mock infected control cells were harvested and processed for small RNA sequencing. Small RNAs between 18 and 35 nt were aligned with antigenome and genome sense rTOSV S, M and L RNA segments. In samples derived from rTOSV-infected PP9ad cells there was a marked increase in small RNAs mapping to each of the rTOSV segments compared to mock-infected counterparts. The majority of the small RNA reads mapping to viral RNA were targeted towards the antigenomic RNA produced during virus replication (Fig 7).

**Fig 8:**
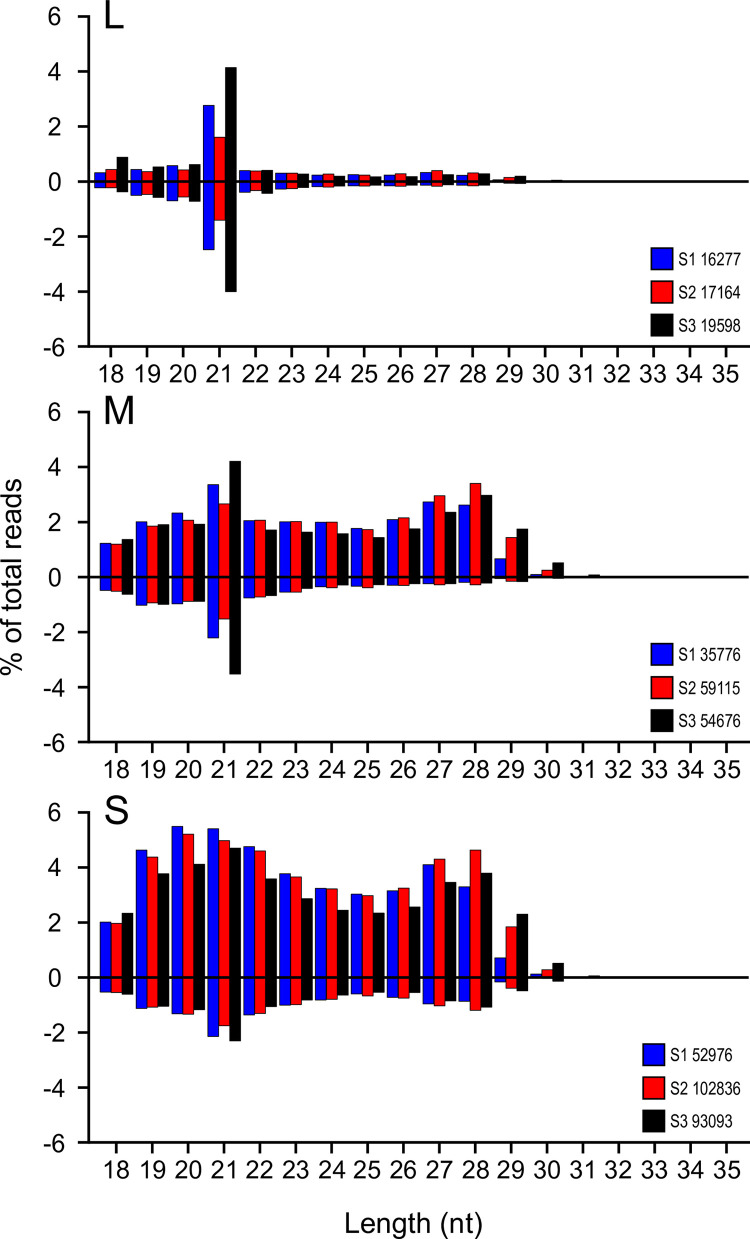
Size distribution of reads mapping to rTOSV L, M and S segments. Read lengths are indicated from 18 to 35 nt; with percentages of total reads per length mapping to the antigenome (positive numbers, y axis) or genome (negative numbers, y axis) indicated. Total read numbers per genome segment from each independent experiment (S1-3) are indicated.

We observed a marked dominance of 21 nt vsiRNAs mapping to the L segment genome or antigenome, over other read sizes. This was also seen for the M segment, but less pronounced for the S segment (Fig 8).

**Fig 9:**
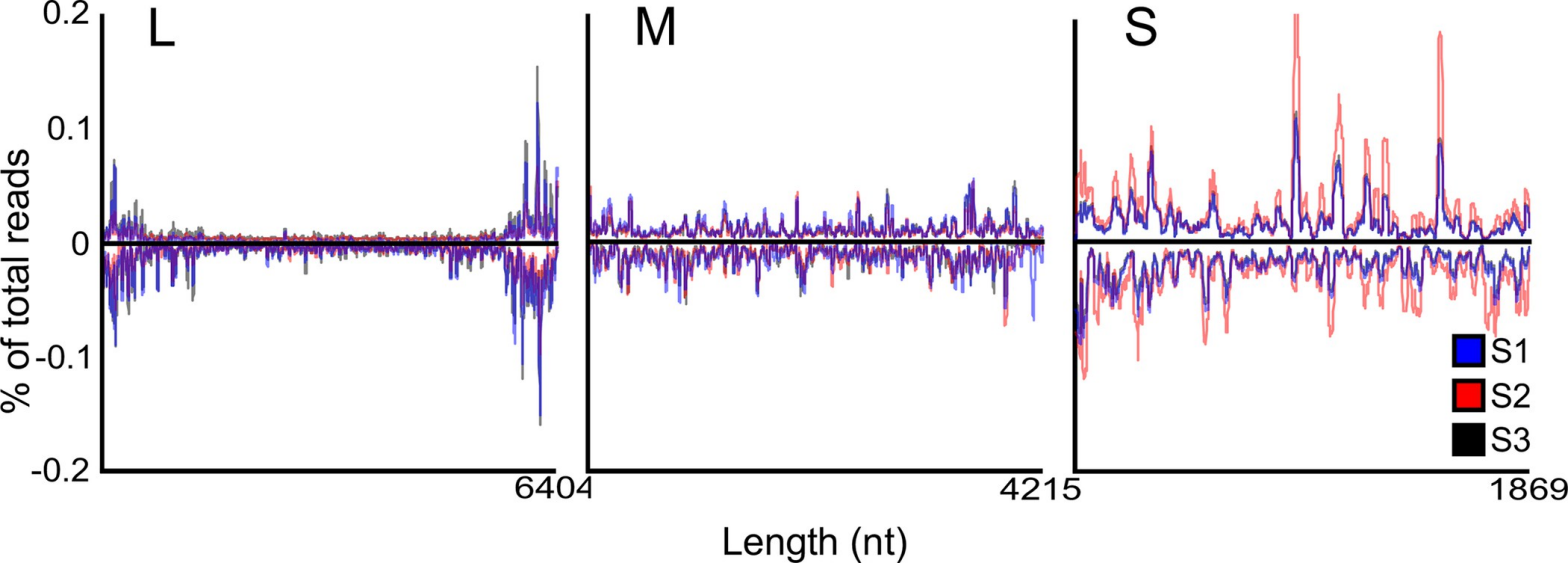
The distribution of 21 nt vsiRNA reads mapped to rTOSV segments. Graphs show the distribution by genomic coverage of 21 nt vsiRNAs along each rTOSV segment length, normalised as a percentage of total reads, per replicate as indicated by colour. Read numbers mapping genome sense are indicated by (-) and antigenome by (+).

Next, we analysed the distribution of the 21 nt vsiRNAs that mapped to genomic or antigenomic sense segment RNAs of rTOSV in order to assess their distribution. For the L segment, these vsiRNAs were concentrated towards the 5’ and 3’ UTRs in both the genomic and antigenomic sense RNAs, with a more even distribution observed for the M and S segment RNAs (Fig 9).

**Fig 10:**
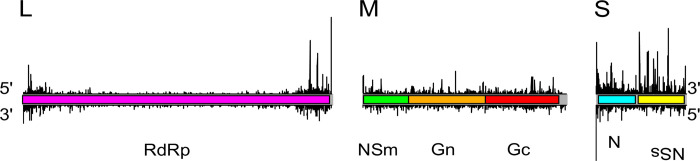
Summary of 21 nt vsiRNA distribution in PP9ad cells in response to rTOSV infection. These graphs show the nucleotide positions for which all three replicates had 21 nt vsiRNAs sequences starting; the data was all scaled across segments by replicate to take differences in read quantity into account and shows the mean of the triplicate. Reads mapping to antigenome on top and genome, below.

As the results were similar over each replicate, we also plotted the start position of 21 nt vsiRNAs reads by segment shared across the replicates (Fig 10) to show their distribution per segment.

### The PP9ad cell line fails to produce vpiRNAs in response to rTOSV infection

**Fig 11:**
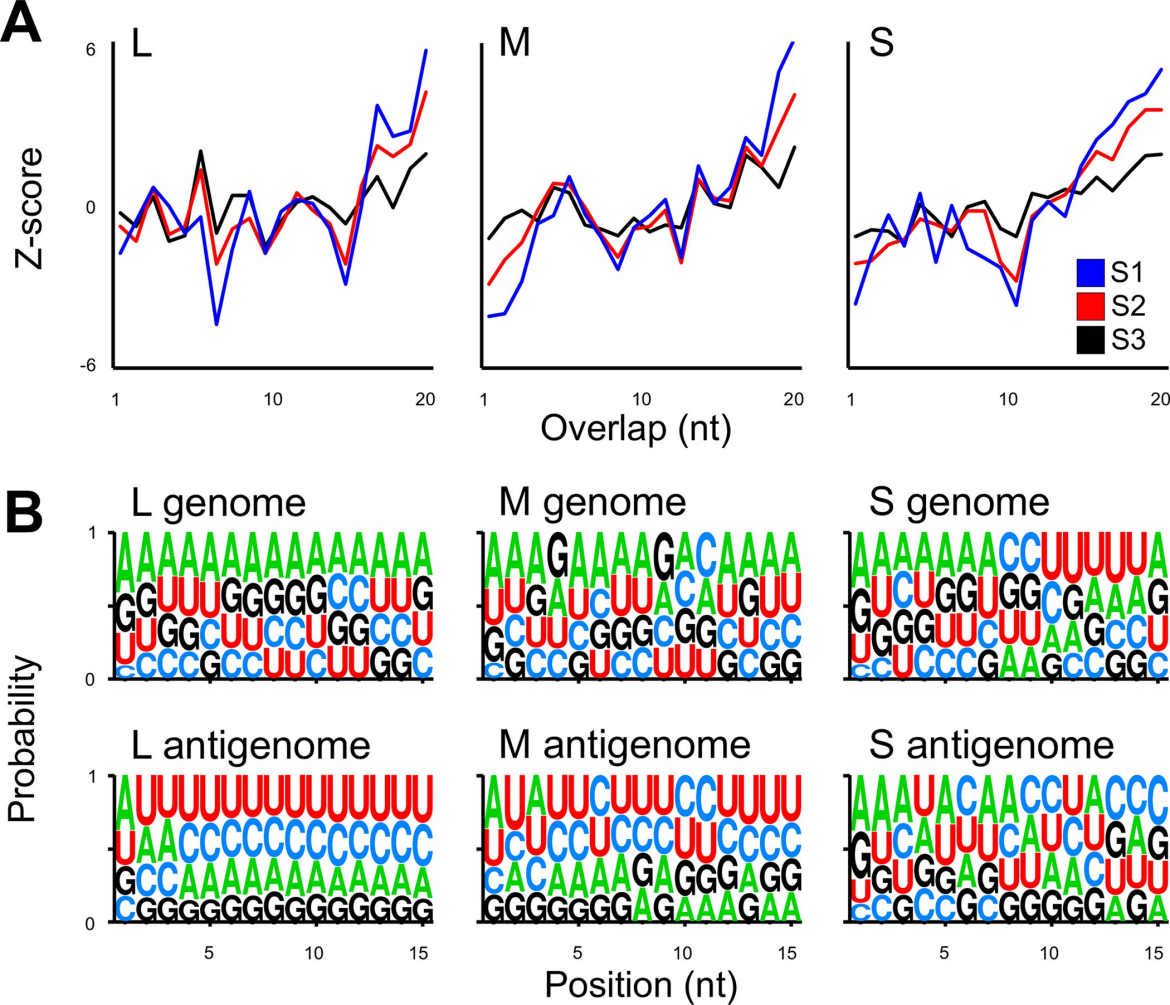
Z-scores and sequence logo analysis of 24–29 nt small RNAs produced in rTOSV-infected PP9ad cells. **(A)** Z-score of combined genomic and antigenomic 24–29 nt small RNAs mapping to rTOSV L, M or S segments. Expected for vpiRNAs would be a prominent peak at the 10 nt overlap position if these were present. Individual replicates are indicated by colour, S1-3. **(B)** Sequence logo analysis, as determined by seqLogo of nucleotide predominance of the first 15 nt of each of 24–29 nt small RNAs (mapping to L, M or S genome or antigenome segments, as indicated) of all replicates combined.

We also investigated the sequence data for the signatures of potential rTOSV-derived vpiRNAs by separately analysing small RNAs 24–29 nt in length. Importantly, vpiRNAs have a characteristic predominance of U(1)/A(10) and 10 nt 5’ overlap within the small RNAs produced in that size range [29]. These characteristic signatures were missing from the small RNAs examined in response to rTOSV infection. Neither a predominance of U in position 1 or A in 10, nor the piRNA specific overlap patterns were observed as shown by overlap and seqLogo [31] analysis (Fig 11). Suggesting that a classical antiviral piRNA response was not induced in rTOSV-infected PP9ad cells.

## Discussion

Here we investigated the RNAi response to rTOSV infection in cells of the *P*. *papatasi* sand fly *in vitro*. These cells produced a robust RNAi response to infection, with small RNAs targeting all segments. Though 21 nt vsiRNAs were detected for all segments, they were dominant for the rTOSV L and M segments. The greater diversity of small RNA reads observed for S segment is intriguing and it may be that copy numbers for each segment or targeting come into play. We were not able to discern any specific signatures or preferences (eg., dominance of nucleotides in given positions, or positions along segments) in vsiRNA sequences. This raises questions on targeting and detection of viral RNA by sand fly Dcr2 homolog(s) and deserves further investigation. Though characterization of putative Dcr2 sequence(s) and development of assay systems for Dcr2 mutants for example, would be required for this. Nonetheless, the presence of 21 nt vsiRNAs is comparable to observations with related RVFV across insect systems [13,14]. The presence of the exo-siRNA pathway in PP9ad cells was also confirmed by our reporter assays. We were able to successfully transfect and express reporter proteins in these cells. Using a dual luciferase reporter system using the PUb promoter in conjunction with luciferase expressing plasmids, dsFFLuc completely abolished FFLuc expression- demonstrating an active RNAi response that can be triggered by dsRNA. We were also able to identify a putative sequence for Ago2 from transcriptomic data; and assembled and confirmed the full length Ago2 sequence from the PP9ad cell line. The functionality and requirement of Ago2 in the dsRNA-mediated exo-siRNA response in these cells was confirmed experimentally. The detection of these small RNAs alongside the importance of Ago2 suggests conservation of the main features of the exo-siRNA pathway as observed for mosquitoes [7–10] also in sand flies, with the absence of vpiRNAs equally in line with previous observations [17]. This raises the question of the relevance of the piRNA pathway in antiviral responses in sand flies. A preliminary investigation of the *P*. *papatasi* transcriptome suggested the presence of putative PIWI proteins, but sequences will need further verification before we can draw final conclusions. Antiviral responses may have evolved differently between sand flies and insects such as mosquitoes. Given the lack of full sequence information we were not able to attempt silencing experiments against any Piwi proteins and assess effects on viral replication. It cannot be excluded that vpiRNAs may have been produced but in too low quantity to be detectable in our analysis. Nonetheless, the data shown here add to our understanding of antiviral responses across arthropods. However, any role of Piwi proteins in this system remains to be investigated. Our data do show a role for the exo-siRNA pathway in responding to rTOSV replication.

The data presented here also indicate the suitability of PP9ad cells for studies of the sand fly antiviral RNAi response, importantly by assessing a sand fly transmitted bunyavirus. Given the relevance of these insects as arbovirus vectors, comparative studies with other sand fly-transmitted arboviruses are facilitated by such a cell system. Importantly, the exo-siRNA pathway of sand flies, as identified in the PP9ad cell line closely resembles that observed in other insects, including mosquitoes which indicates the functional and evolutionary importance, and conservation of this important antiviral response across arthropods.

## Supporting information

S1 Method Production protocol for PP9ad cells. (DOCX)

S1 Table This table shows primers used in this study. Further information is given at each primer set.
(DOCX)

S2 Table This table shows accession numbers for Ago2 protein sequences from insect species that were used for multiple sequence alignment and phylogenetic analysis, as indicated. (DOCX)

## References

[1] Ayhan N, Charrel RN. An update on Toscana virus distribution, genetics, medical and diagnostic aspects. Clin Microbiol Infect. 2020;26(8):1017–23. Epub 2020/01/07. doi: 10.1016/j.cmi.2019.12.015 . 31904562

[2] Charrel RN, Bichaud L, de Lamballerie X. Emergence of Toscana virus in the mediterranean area. World J Virol. 2012;1(5):135–41. Epub 2013/11/01. doi: 10.5501/wjv.v1.i5.135 ; PubMed Central PMCID: PMC3782275. 24175218 PMC3782275

[3] Elliott RM, Brennan B. Emerging phleboviruses. Curr Opin Virol. 2014;5:50–7. Epub 2014/03/13. doi: 10.1016/j.coviro.2014.01.011 ; PubMed Central PMCID: PMC4031632. 24607799 PMC4031632

[4] Wuerth JD, Weber F. Phleboviruses and the Type I Interferon Response. Viruses. 2016;8(6). Epub 2016/06/25. doi: 10.3390/v8060174 ; PubMed Central PMCID: PMC4926194. 27338447 PMC4926194

[5] Woelfl F, Leger P, Oreshkova N, Pahmeier F, Windhaber S, Koch J, et al. Novel Toscana Virus Reverse Genetics System Establishes NSs as an Antagonist of Type I Interferon Responses. Viruses. 2020;12(4). Epub 2020/04/09. doi: 10.3390/v12040400 ; PubMed Central PMCID: PMC7232479. 32260371 PMC7232479

[6] Alexander AJT, Confort MP, Desloire S, Dunlop JI, Kuchi S, Sreenu VB, et al. Development of a Reverse Genetics System for Toscana Virus (Lineage A). Viruses. 2020;12(4). Epub 2020/04/11. doi: 10.3390/v12040411 ; PubMed Central PMCID: PMC7232365. 32272808 PMC7232365

[7] Blair CD, Olson KE. The role of RNA interference (RNAi) in arbovirus-vector interactions. Viruses. 2015;7(2):820–43. Epub 2015/02/19. doi: 10.3390/v7020820 ; PubMed Central PMCID: PMC4353918. 25690800 PMC4353918

[8] Olson KE, Blair CD. Arbovirus-mosquito interactions: RNAi pathway. Curr Opin Virol. 2015;15:119–26. Epub 2015/12/03. doi: 10.1016/j.coviro.2015.10.001 ; PubMed Central PMCID: PMC4765169. 26629932 PMC4765169

[9] Samuel GH, Adelman ZN, Myles KM. Antiviral Immunity and Virus-Mediated Antagonism in Disease Vector Mosquitoes. Trends Microbiol. 2018;26(5):447–61. Epub 2018/02/06. doi: 10.1016/j.tim.2017.12.005 ; PubMed Central PMCID: PMC5910197. 29395729 PMC5910197

[10] Tikhe CV, Dimopoulos G. Mosquito antiviral immune pathways. Dev Comp Immunol. 2021;116:103964. Epub 2020/12/11. doi: 10.1016/j.dci.2020.103964 . 33301792

[11] Gestuveo RJ, Parry R, Dickson LB, Lequime S, Sreenu VB, Arnold MJ, et al. Mutational analysis of Aedes aegypti Dicer 2 provides insights into the biogenesis of antiviral exogenous small interfering RNAs. PLoS Pathog. 2022;18(1):e1010202. Epub 2022/01/07. doi: 10.1371/journal.ppat.1010202 ; PubMed Central PMCID: PMC8769306. 34990484 PMC8769306

[12] Varjak M, Leggewie M, Schnettler E. The antiviral piRNA response in mosquitoes? J Gen Virol. 2018;99(12):1551–62. Epub 2018/10/30. doi: 10.1099/jgv.0.001157 . 30372405

[13] Dietrich I, Jansen S, Fall G, Lorenzen S, Rudolf M, Huber K, et al. RNA Interference Restricts Rift Valley Fever Virus in Multiple Insect Systems. mSphere. 2017;2(3). Epub 2017/05/13. doi: 10.1128/mSphere.00090-17 ; PubMed Central PMCID: PMC5415632. 28497117 PMC5415632

[14] Leger P, Lara E, Jagla B, Sismeiro O, Mansuroglu Z, Coppee JY, et al. Dicer-2- and Piwi-mediated RNA interference in Rift Valley fever virus-infected mosquito cells. J Virol. 2013;87(3):1631–48. Epub 2012/11/24. doi: 10.1128/JVI.02795-12 ; PubMed Central PMCID: PMC3554164. 23175368 PMC3554164

[15] Pitaluga AN, Mason PW, Traub-Cseko YM. Non-specific antiviral response detected in RNA-treated cultured cells of the sandfly, Lutzomyia longipalpis. Dev Comp Immunol. 2008;32(3):191–7. Epub 2007/08/21. doi: 10.1016/j.dci.2007.06.008 . 17706772

[16] Martins-da-Silva A, Telleria EL, Batista M, Marchini FK, Traub-Cseko YM, Tempone AJ. Identification of Secreted Proteins Involved in Nonspecific dsRNA-Mediated Lutzomyia longipalpis LL5 Cell Antiviral Response. Viruses. 2018;10(1). Epub 2018/01/19. doi: 10.3390/v10010043 ; PubMed Central PMCID: PMC5795456. 29346269 PMC5795456

[17] Ferreira FV, Aguiar E, Olmo RP, de Oliveira KPV, Silva EG, Sant’Anna MRV, et al. The small non-coding RNA response to virus infection in the Leishmania vector Lutzomyia longipalpis. PLoS Negl Trop Dis. 2018;12(6):e0006569. Epub 2018/06/05. doi: 10.1371/journal.pntd.0006569 ; PubMed Central PMCID: PMC6002125. 29864168 PMC6002125

[18] Rozo-Lopez P, Drolet BS, Londono-Renteria B. Vesicular Stomatitis Virus Transmission: A Comparison of Incriminated Vectors. Insects. 2018;9(4). Epub 2018/12/14. doi: 10.3390/insects9040190 ; PubMed Central PMCID: PMC6315612. 30544935 PMC6315612

[19] Hilton L, Moganeradj K, Zhang G, Chen YH, Randall RE, McCauley JW, et al. The NPro product of bovine viral diarrhea virus inhibits DNA binding by interferon regulatory factor 3 and targets it for proteasomal degradation. J Virol. 2006;80(23):11723–32. Epub 2006/09/15. doi: 10.1128/JVI.01145-06 ; PubMed Central PMCID: PMC1642611. 16971436 PMC1642611

[20] Mottram TJ, Li P, Dietrich I, Shi X, Brennan B, Varjak M, et al. Mutational analysis of Rift Valley fever phlebovirus nucleocapsid protein indicates novel conserved, functional amino acids. PLoS Negl Trop Dis. 2017;11(12):e0006155. Epub 2017/12/22. doi: 10.1371/journal.pntd.0006155 ; PubMed Central PMCID: PMC5764413. 29267287 PMC5764413

[21] Tesh RB, Modi GB. Development of a continuous cell line from the sand fly Lutzomyia longipalpis (Diptera: Psychodidae), and its susceptibility to infection with arboviruses. J Med Entomol. 1983;20(2):199–202. Epub 1983/03/30. doi: 10.1093/jmedent/20.2.199 . 6842527

[22] Vasilakis N, Widen S, Mayer SV, Seymour R, Wood TG, Popov V, et al. Niakha virus: a novel member of the family Rhabdoviridae isolated from phlebotomine sandflies in Senegal. Virology. 2013;444(1–2):80–9. Epub 2013/06/19. doi: 10.1016/j.virol.2013.05.035 ; PubMed Central PMCID: PMC3755043. 23773405 PMC3755043

[23] Grabherr MG, Haas BJ, Yassour M, Levin JZ, Thompson DA, Amit I, et al. Full-length transcriptome assembly from RNA-Seq data without a reference genome. Nat Biotechnol. 2011;29(7):644–52. Epub 2011/05/17. doi: 10.1038/nbt.1883 ; PubMed Central PMCID: PMC3571712. 21572440 PMC3571712

[24] Haas BJ, Papanicolaou A, Yassour M, Grabherr M, Blood PD, Bowden J, et al. De novo transcript sequence reconstruction from RNA-seq using the Trinity platform for reference generation and analysis. Nat Protoc. 2013;8(8):1494–512. Epub 2013/07/13. doi: 10.1038/nprot.2013.084 ; PubMed Central PMCID: PMC3875132. 23845962 PMC3875132

[25] Petrella V, Aceto S, Musacchia F, Colonna V, Robinson M, Benes V, et al. De novo assembly and sex-specific transcriptome profiling in the sand fly Phlebotomus perniciosus (Diptera, Phlebotominae), a major Old World vector of Leishmania infantum. BMC Genomics. 2015;16:847. Epub 2015/10/27. doi: 10.1186/s12864-015-2088-x ; PubMed Central PMCID: PMC4619268. 26493315 PMC4619268

[26] Varjak M, Dietrich I, Sreenu VB, Till BE, Merits A, Kohl A, et al. Spindle-E Acts Antivirally Against Alphaviruses in Mosquito Cells. Viruses. 2018;10(2). Epub 2018/02/22. doi: 10.3390/v10020088 ; PubMed Central PMCID: PMC5850395. 29463033 PMC5850395

[27] Varjak M, Donald CL, Mottram TJ, Sreenu VB, Merits A, Maringer K, et al. Characterization of the Zika virus induced small RNA response in Aedes aegypti cells. PLoS Negl Trop Dis. 2017;11(10):e0006010. Epub 2017/10/19. doi: 10.1371/journal.pntd.0006010 ; PubMed Central PMCID: PMC5667879. 29040304 PMC5667879

[28] Anderson MA, Gross TL, Myles KM, Adelman ZN. Validation of novel promoter sequences derived from two endogenous ubiquitin genes in transgenic Aedes aegypti. Insect Mol Biol. 2010;19(4):441–9. Epub 2010/05/12. doi: 10.1111/j.1365-2583.2010.01005.x ; PubMed Central PMCID: PMC3605713. 20456509 PMC3605713

[29] Varjak M, Maringer K, Watson M, Sreenu VB, Fredericks AC, Pondeville E, et al. Aedes aegypti Piwi4 Is a Noncanonical PIWI Protein Involved in Antiviral Responses. mSphere. 2017;2(3). Epub 2017/05/13. doi: 10.1128/mSphere.00144-17 ; PubMed Central PMCID: PMC5415634. 28497119 PMC5415634

[30] Taylor SC, Nadeau K, Abbasi M, Lachance C, Nguyen M, Fenrich J. The Ultimate qPCR Experiment: Producing Publication Quality, Reproducible Data the First Time. Trends Biotechnol. 2019;37(7):761–74. Epub 2019/01/19. doi: 10.1016/j.tibtech.2018.12.002 . 30654913

[31] Bembom O, Ivanek R. seqLogo: Sequence logos for DNA sequence alignments. R package version 1.62.0. 2022.

