# Supplementary material for: Characterisation of the antiviral RNA interference response to Toscana virus in sand fly cells
Source: PLoS Pathog. 2023 Mar 30;19(3):e1011283. doi: 10.1371/journal.ppat.1011283 (PMC10112792; doi:10.1371/journal.ppat.1011283)
Supplement: S1 Method — (DOCX) [file ppat.1011283.s001.docx]

**S1 Method.** Production protocol for PP9ad cells.

Initially as received the PP9 cells were supplied, and recommended to grow in, a 1:1 mix of Mitsuhashi & Maramorosch medium (M & M) and Schneiders Drosophila medium (Gibco, Waltham, MA, USA) (without L-Glutamine) supplemented with 20% FCS (Gibco, Waltham, MA, USA). The cells largely grew as suspended multicellular ball like clumps and required constant breaking apart. This additionally caused issues with the cells being ripped apart during the mixing.

To improve their suitability for cell culture-based experiments and improve adherence, Pluronic F68 (Sigma-Aldrich, St. Louis, MO, USA) was initially added to the media to try and prevent both clumping and shearing but this had very little effect. Maintaining the addition of Pluronic F68 the cells were vigorously split and the mix of M & M:Schneiders Drosophila medium changed in 10% increments to 100% Schneiders Drosophila medium. This prevented clumping but the cells were still in suspension, and still unamenable to transfection.

The cells were then split into a range of media compositions in 100% Leibovitz's L-15 medium (Gibco, Waltham, MA, USA): L-15 + TPB (Gibco, Waltham, MA, USA), or + L-Glutamine (200mM) (Gibco, Waltham, MA, USA) and + both L-Glutamine and TPB. Each flask was repeated for a range of FCS concentrations, from 20% to 0%. The cells were left for 2 weeks before media change to remove suspension cells. After a further 2 weeks the condition with L-15 + L-Glutamine and TPB with 10% FCS had the fastest growing, adherent cells. After passaging (with suspension cells removed at media change) and testing, as these cells were transfectable and no further optimisation was performed.
