# Supplementary material for: Characterisation of the antiviral RNA interference response to Toscana virus in sand fly cells
Source: PLoS Pathog. 2023 Mar 30;19(3):e1011283. doi: 10.1371/journal.ppat.1011283 (PMC10112792; doi:10.1371/journal.ppat.1011283)
Supplement: S1 Table — Further information is given at each primer set. (DOCX) [file ppat.1011283.s002.docx]

**S1 Table**. This table shows primers used in this study. Further information is given at each primer set.

| **Primers used for generation of plasmids** | |
| --- | --- |
| pPUb_Fw | TTCTAGAGTCGGGGCGGC |
| pPUb_Rv | GGTTGAAATCTCTGTTGAGCAG |
| pRL_RLuc_Fw | TCAACAGAGATTTCAACCATGACTTCGAAAGTTTATGATCC |
| pRL_RLuc_Rv | GCCCCGACTCTAGAATTATTGTTCATTTTTGAGAACTCG |
| **Primers used for amplification and sequencing of PP9ad Ago2 cDNA** | |
| PPap Ago2 Fw | ATGGATAATCCAGATAAAAAGAAGAGG |
| PPap Ago2 Rv | TTAGACGAAAAACATTGGATG |
| Ago2-Rev_1 | CCTATCGGGCTCATTTCTGA |
| Ago2-Rev_2 | CATTTCCTGGTCAAATTTTCG |
| Ago2-Rev_3 | CATGCATCTTCAAATGCTCC |
| Ago2-For_1 | GCCATGTCCAGGAAGAAGAC |
| Ago2-For_2 | AAGATAATATCAGCCGGAAAAGC |
| Ago2-For_3 | ATGTAGCACCTACCTATTATGCCC |
| **Primers for generation of dsRNA targeting Ago2, the primers contain a T7 promoter at both the 5’ and 3’** | |
| dsAgo2 Fw | *GTAATACGACTCACTATAGGG*GAAATCCCTTCAGATAAAAGAAGGTGATC |
| dsAgo2 Rv | *GTAATACGACTCACTATAGGG*GCTATGTCATAATGATAGGCCACATCGAC |
| **Ago2 5’ RACE Reverse Transcription primers** | |
| TSO (DNA/RNA hybrid primer) | *GCTAATCATTGCAAGCAGTGGTATCAACGCAGAGTACATRGRGRG* |
| AGO2_5'RACE_Rv | CCTATCGGGCTCATTTCTGAAAGCTTGCC |
| **Ago2 5’ RACE RT primers, PCR 1: amplification from cDNA** | |
| TSOoligo (PCR from TSO) | CATTGCAAGCAGTGGTATCAACGC |
| AGO2 5'RACE PCR Rv | *GTCCACCAGGAGGCAGGGTCAGC* |
| **Primers for sequencing of Ago2 5’ RACE product** | |
| AGO2_5'_Rv1 | TCGACGATTCCTCTTCTTTTT |
| AGO2_5'_Rv2 | TCCTCTCCTCAGCAGGCTT |
| AGO2_5'_Rv3 | TAGGCTCGCTGGTCTGCT |
| AGO2_5'_Rv4 | CCTCCAGGTACGCTCATAGG |
| AGO2_5'_Rv5 | GATCGTTGCATCTGCTGAG |
| AGO2_5'_Rv6 | TTGCTGTTGCATCTTCTGCT |
| **QRT-PCR primers for Sod1 and Ago2** | |
| ***Sod1*** |  |
| 5’ PRIMER | GGCCACTCAGAGAAATCTGC |
| 3’ PRIMER | ACTACAATCCCCATGGCAAG |
| ***Ago2*** |  |
| 5’ PRIMER | GTGCTTCATGTTGGTCCGAA |
| 3’ PRIMER | GCTACGCGTCTGATTGTCTG |
