# Supplementary material for: Characterisation of the antiviral RNA interference response to Toscana virus in sand fly cells
Source: PLoS Pathog. 2023 Mar 30;19(3):e1011283. doi: 10.1371/journal.ppat.1011283 (PMC10112792; doi:10.1371/journal.ppat.1011283)
Supplement: S2 Table — (DOCX) [file ppat.1011283.s003.docx]

**S2 Table**. This table shows accession numbers for Ago2 protein sequences from insect species that were used for multiple sequence alignment and phylogenetic analysis, as indicated.

| **Accession** | **Species name** |
| --- | --- |
| XP_011493002 | *Aedes aegypti* |
| XP_029716852 | *Aedes albopictus* |
| XP_050095446 | *Anopheles aquasalis* |
| XP_040163957 | *Anopheles arabiensis* |
| XP_040233692 | *Anopheles coluzzii* |
| ETN67307 | *Anopheles darlingi* |
| XP_049298709 | *Anopheles funestus* |
| AGAP029751 | *Anopheles gambiae* |
| XP_050080093 | *Anopheles maculipalpis* |
| XP_041782936 | *Anopheles merus* |
| KFB40608 | *Anopheles sinensis* |
| XP_035892675 | *Anopheles stephensi* |
| XP_019846276 | *Bactrocera dorsalis* |
| XP_018793217 | *Bactrocera latifrons* |
| XP_050337572 | *Bactrocera neohumeralis* |
| XP_036216615 | *Bactrocera oleae* |
| XP_039963203 | *Bactrocera tryoni* |
| XP_037046508 | *Bradysia coprophila* |
| KAG4066948 | *Bradysia odoriphaga* |
| XP_020716165 | *Ceratitis capitata* |
| CRL06019 | *Clunio marinus* |
| XP_031627620 | *Contarinia nasturtii* |
| XP_038118188 | *Culex quinquefasciatus* |
| XP_034111510 | *Drosophila albomicans* |
| XP_001956678 | *Drosophila ananassae* |
| XP_017863326 | *Drosophila arizonae* |
| XP_016958952 | *Drosophila biarmipes* |
| XP_017103997 | *Drosophila bipectinata* |
| XP_017112543 | *Drosophila elegans* |
| XP_001973098 | *Drosophila erecta* |
| XP_041674135 | *Drosophila eugracilis* |
| XP_017053202 | *Drosophila ficusphila* |
| SPP87304 | *Drosophila guanche* |
| XP_030081381 | *Drosophila hydei* |
| AHZ92157 | *Drosophila immigrans* |
| XP_034483721 | *Drosophila innubila* |
| XP_017034167 | *Drosophila kikkawai* |
| XP_033161911 | *Drosophila mauritiana* |
| FBgn0087035 | *Drosophila melanogaster* |
| XP_002007747 | *Drosophila mojavensis* |
| TDG42828 | *Drosophila navojoa* |
| XP_030568436 | *Drosophila novamexicana* |
| XP_041449380 | *Drosophila obscura* |
| XP_016984986 | *Drosophila rhopaloa* |
| XP_039489637 | *Drosophila santomea* |
| XP_002030605 | *Drosophila sechellia* |
| XP_020798228 | *Drosophila serrata* |
| XP_016031993 | *Drosophila simulans* |
| XP_034658415 | *Drosophila subobscura* |
| XP_037721025 | *Drosophila subpulchrella* |
| XP_036670568 | *Drosophila suzukii* |
| XP_017010239 | *Drosophila takahashii* |
| XP_043646402 | *Drosophila teissieri* |
| XP_032291133 | *Drosophila virilis* |
| XP_023032415 | *Drosophila willistoni* |
| XP_002095171 | *Drosophila yakuba* |
| ALC79939 | *Episyrphus balteatus* |
| ALC79938 | *Eristalis pertinax* |
| XP_037892446 | *Glossina fuscipes* |
| CAD7081280 | *Hermetia illucens* |
| KAI8122196 | *Lucilia cuprina* |
| XP_037807749 | *Lucilia sericata* |
| LLOJ006148 | *Lutzomyia longipalpis* |
| OP744464 | *Phlebotomus papatasi* |
| XP_036334172 | *Rhagoletis pomonella* |
| XP_017474419 | *Rhagoletis zephyria* |
| ALC79937 | *Tabanus bromius* |
| XP_037940129 | *Teleopsis dalmanni* |
| XP_028894702 | *Zeugodacus cucurbitae* |
| QUP51772.1 | *Locusta migratoria* |
